# Co-Expression and Combined Prognostic Value of CSPG4 and PDL1 in *TP53*-Aberrant Triple-Negative Breast Cancer

**DOI:** 10.3389/fonc.2022.804466

**Published:** 2022-02-24

**Authors:** Zhe-Yu Hu, Chanjuan Zheng, Jianbo Yang, Siyu Ding, Can Tian, Ning Xie, Lian Xue, Muyao Wu, Shujun Fu, Zhouzhou Rao, Matthew A. Price, James B. McCarthy, Quchang Ouyang, Jizhen Lin, Xiyun Deng

**Affiliations:** ^1^ Hunan Cancer Hospital and the Affiliated Cancer Hospital of Xiangya Medical School, Central South University, Changsha, China; ^2^ Department of Breast Cancer Medical Oncology, Hunan Cancer Hospital, Changsha, China; ^3^ Department of Breast Cancer Medical Oncology, the Affiliated Cancer Hospital of Xiangya Medical School, Central South University, Changsha, China; ^4^ Key Laboratory of Model Animals and Stem Cell Biology in Hunan Province, Department of Pathophysiology, Hunan Normal University School of Medicine, Changsha, China; ^5^ Key Laboratory of Translational Cancer Stem Cell Research, Hunan Normal University, Changsha, China; ^6^ Department of Laboratory Medicine and Pathology and Comprehensive Cancer Center, University of Minnesota, Minneapolis, MN, United States; ^7^ The Cancer Center, Union Hospital, Fujian Medical Center, Fuzhou, China; ^8^ Department of Otolaryngology, Cancer Center, University of Minnesota Medical School, Minnesota, MN, United States

**Keywords:** triple-negative breast cancer, *TP53* aberration, chondroitin sulfate proteoglycan 4, programmed cell death ligand 1, prognosis

## Abstract

**Background:**

In triple-negative breast cancer (TNBC), PDL1/PD1-directed immunotherapy is effective in less than 20% of patients. In our preliminary study, we have found CSPG4 to be highly expressed together with PDL1 in TNBCs, particularly those harboring *TP53* aberrations. However, the clinical implications of co-expressed CSPG4 and PDL1 in TNBCs remain elusive.

**Methods:**

A total of 85 advanced TNBC patients treated in the Hunan Cancer Hospital between January 2017 and August 2019 were recruited. The expressions of CSPG4 and PDL1 in TNBC tissues were investigated using immunohistochemistry (IHC). The RNA-seq dataset from the TCGA-BRCA project was further used to analyze the mRNA expression of CSPG4 and PDL1 in *TP53*-aberrant TNBCs. Cox proportional hazards model and Kaplan–Meier curves with Logrank test was used to analyze the effects of CSPG4 and PDL1 on survival. TNBC cell lines were further used to investigate the molecular mechanism that were involved.

**Results:**

*TP53* aberrations occurred in more than 50% of metastatic TNBCs and were related to higher tumor mutation burden (TMB). In TCGA-BRCA RNA-seq dataset analysis, both CSPG4 and PDL1 levels were high in TNBCs, especially in *TP53*-aberrant TNBCs. IHC assay showed nearly 60% of advanced TNBCs to be CSPG4-positive and about 25% to be both CSPG4-positive and PDL1-positive. The levels of CSPG4 and PDL1 were high in TNBC cell lines as revealed by flow cytometry and immunoblotting compared with non-TNBC cells. Univariate Cox regression analysis indicated that CSPG4 positivity was a significant risk factor for progression-free survival in metastatic TNBCs, with a hazard ratio (HR) of 2.26 (*P* = 0.05). KM curves with Logrank test also identified high level of CSPG4 as a significant risk factor for overall survival in advanced breast cancers in TCGA-BRCA samples (*P* = 0.02). The immunoblotting assays showed that EMT-related pathways were involved in CSPG4-mediated invasion.

**Conclusions:**

CSPG4 expression level is associated with PDL1 positivity in *TP53*-aberrant TNBC cells. Patients with CSPG4 expression have poor treatment response and poor overall survival. Co-expressed CSPG4 and PDL1 may have an important prognostic value and provide new therapeutic targets in TNBC patients. CSPG4 might mediate tumor invasion and PDL1 overexpression through EMT-related pathway.

## Introduction

Breast cancer is the most common malignancy threatening the health of women around the world. Triple-negative breast cancer (TNBC) is characterized by negative expression of the hormone receptors [i.e., estrogen receptor (ER) and progesterone receptor (PR)] and the human epidermal growth factor receptor 2 (HER2), accounting for about 10–20% of all breast cancer cases. According to the St. Gallen consensus, the prognosis of TNBC is the worst among all subtypes of breast cancer (1). At present, single or combined chemotherapy is the mainstay of treatment for late-stage TNBC. However, after multiline chemotherapy, drug resistance occurs and the disease progresses rapidly. The median overall survival (OS) of patients with metastatic TNBC (mTNBC) is about 14.5 months (2), which is much shorter than that of luminal-type patients (42.9 months) and HER2-enriched patients (50.1 months). It is well known that the expression of PDL1 (also known as CD274) in breast cancer is associated with large tumor size, high grade, and high proliferation (3, 4). Although immune checkpoint inhibition using anti-PDL1 antibody, e.g., atezolizumab, in combination with chemotherapy has shown great promise in TNBC (5, 6), a majority of TNBC patients still do not benefit from PDL1-targeted immunotherapy. Therefore, challenges remain, particularly regarding the need for improvement of the therapeutic efficacy.

It has been demonstrated that *TP53* aberrations are prevalent in TNBC, with roughly 40–62% of patients having *TP53* aberrations, followed by *PIK3CA* aberrations in 10% of patients and aberrations of other genes, namely, *Rb1*, *PTEN*, *BRCA2*, *erbB2/3*, and *BRAF* in 7–9% of patients (7). Cancer cells with DNA damage induced by chemotherapy would be blocked by the p53 protein and enter the apoptosis program. The ATM/Chk2-p53 signaling pathway plays a critical role in cell cycle arrest, DNA repair and apoptosis induced by DNA-damaging agents (8). *TP53* aberrations are associated with poor treatment response and prognosis in breast cancers (9, 10). Breast cancer patients with *TP53* aberrations and particularly TNBC patients are more likely to be resistant to anti-cancer treatment (11–13). Gene abnormalities related to *TP53* aberrations in TNBC, such as 9p24.1 amplification and *PIK3CA* gene mutation, abnormal PI3K, ErbB1/EGFR, MUC1, Alix, and PARP-GSK3β signaling pathways, could alter immunogenicity (14–16) and are known to be associated with PDL1/PD1 abnormalities (17).

It has been demonstrated that chondroitin sulfate proteoglycan 4 (CSPG4), a scaffold protein composed of chondroitin sulfate and proteoglycan with multiple cancer-promoting functions, is overexpressed in TNBC (18). CSPG4 promotes tumor cell proliferation, angiogenesis, drug resistance, immune escape, and radiation resistance (19–21). CSPG4 binds to a variety of kinases and extracellular factors to mediate the activation of multiple signaling pathways (22). In TNBC, it has been demonstrated that CSPG4 binds to PDL1 on the cell surface. In our preliminary study, we found that CSPG4 and PDL1 are co-expressed in TNBC tissues. However, the clinical implications, i.e., the value of these co-expressed CSPG4 and PDL1 molecules as prognostic predictors in TNBC are not known.

As mentioned above, a majority of TNBC patients do not benefit from immune checkpoint-based immunotherapy. Therefore, increasing the sensitivity to immune checkpoint blockade through exploring new molecules, e.g., those that interact with the PDL1/PD1 axis, is an urgently unmet task. In this study, through next-generation sequencing and tumor mutation burden analysis, we found that CSPG4 was highly expressed together with PDL1 in TNBCs, particularly in those harboring *TP53* aberrations. We also investigated the clinical implications of CSPG4 and PDL1 in TNBC patients by analyzing on-line databases and found that co-expression of CSPG4 and PDL1 has important prognostic value in TNBCs. Overall, our study suggests that co-targeting CSPG4 and PDL1 in advanced TNBC might be a novel strategy to improve the therapeutic efficacy of PDL1-based immune checkpoint blockade.

## Methods

### Study Design and Specimens

This study included 85 recurrent and mTNBC patients treated in the Hunan Cancer Hospital between January 2017 and August 2019. The inclusion criteria were: 1) pathologically confirmed diagnosis of breast cancer; 2) negative expression of ER and PR confirmed by immunohistochemistry (IHC); and 3) negative expression/amplification of HER2 confirmed by IHC/FISH. The exclusion criteria were: 1) multiple primary tumors (≥2); and 2) no measurable invasive breast cancer tissue. The clinicopathological characteristics of the enrolled patients are listed in **Supplementary Table 1**. All molecular pathological data were confirmed by three experienced pathologists. This study was approved by the Ethics Committee of Hunan Cancer Hospital.

### Next-Generation Sequencing and Tumor Mutation Burden Analysis

Among the 85 TNBC patients, 52 voluntarily received circulating tumor DNA (ctDNA) evaluations. Next-generation sequencing (NGS) of ctDNA samples was performed according to our previously published method (9). TMB, which was expressed as the somatic mutations per mega-base (Mb), was calculated from whole exome sequencing data or big gene panels (23, 24). TMB analysis interrogated single nucleotide variants (SNVs) and small INDELs with the variant allele frequency (VAF) ≥3%.

### Transcriptome Profiling of TCGA-BRCA Dataset

This study firstly used the transcriptome dataset of the TCGA-BRCA project (RNA-seq dataset) from the cancer coordination dataset supported by the National Cancer Institute (NCI)—Cancer Genome Atlas. In the RNA-seq dataset, the gene expression level was recorded as the number of fragments per kilobase of exon model per million reads mapped (FPKM). More than 36,218 genes were identified in the HGNC (HUGO (Human Genome Organization) Gene Nomenclature Committee) database by using the Bioconductor “org.HS.eg.db” package.

### TIMER Database Analysis

CSPG4 and PDL1 mRNA expression levels in different types of human cancers and subtypes of breast cancer were analyzed *via* the Tumor Immune Estimation Resource 2.0 (TIMER2.0) database (http://timer.cistrome.org/) (25). Box plots were generated by the Gene DE module to display the distributions of CSPG4 and PDL1 mRNA expression levels. The statistical significance computed by the Wilcoxon test was annotated by the number of stars (*P <0.05, **P <0.01, ***P <0.001).

### Gene Set Enrichment Analysis

Functional enrichment was conducted using Gene Set Enrichment Analysis (GSEA) (https://www.gsea-msigdb.org/gsea/msigdb/index.jsp) to explore whether identified sets of genes showed significant differential expression between the high and low expression groups (26). Gene set permutations were conducted 1,000 times for each analysis. Gene sets with *P <*0.05 and false discovery rate (FDR) <0.25 were considered as enriched.

### STRING Database Analysis

Search tool for retrieval of interacting genes/proteins (STRING) database was applied to evaluate the protein-protein interaction (PPI) network (27). The PPI network between CSPG4 or PDL1 and their correlated proteins was constructed by using the interaction database platform STRING v.11.0 (https://string-db.org/). The species was set to “*Homo sapiens*”, and other parameters were set to default.

### Immunohistochemical (IHC) Analysis

All tissue samples were stained with hematoxylin and eosin, and the presence of invasive breast cancer cells was confirmed by microscopic examination. The protein levels of PDL1 and CSPG4 were assessed by IHC. The IHC steps were as follows: 1) the sections were de-waxed and rehydrated with xylene and alcohol, respectively; 2) the sections were incubated with anti-PDL1 or anti-CSPG4 antibody at 4°C overnight, followed by horseradish peroxidase (HRP)-conjugated secondary antibody incubation and coloration; 3) the stained cells were analyzed microscopically and the positive rate of the stained tumor cells was quantified using the Image-Pro Plus software (Media Controlnetics, Maryland, USA).

According to the clinicopathological diagnostic criteria, PDL1 positivity was defined as the percentage of tumor cells or tumor-infiltrating lymphocytes (TILs) with membranous PDL1 expression ≥1% (28). The positive expression of CSPG4 was brownish yellow or brown and located in the membrane or cytoplasm. The expression level of CSPG4 was scored according to the staining density (no staining scored 0, light brown scored 1 and dark brown scored 2) and the percentage of positive cells (0% scored 0, 1–25% scored 1, 26–50% scored 2, 51–75% scored 3, and >75% scored 4). The total score of CSPG4 was a combination of the staining intensity and the percentage of positive cells, with a total score ≤3 and ≥4 defined as low and high expression, respectively (29).

### CSPG4 Knockdown by CRISPR/CAS9 in TNBC SUM149 Cells

The guide RNAs used to make the CSPG4-CRISPR cells were 5’-CGAGCGCGGCTCTGCTCCTG-3’ and 5’-AGAGACCTGGAGACACCAGG-3’. Both guide RNA plasmids were co-transfected with a plasmid expressing the CAS9 enzyme (pT3.5 Caggs-FLAG-hCas9) and also two plasmids for puromycin and GFP selection, pcDNA-PB7 and pPB SB-CG-LUC-GFP (Puro)(+CRE). Mock cell line was transfected with selection plasmids only (pcDNA-PB7 and pPB SB-CG-LUC-GFP (Puro)(+CRE)) and selected by puromycin-containing medium (0.6 μg/ml). Single cell-derived colonies were expanded and screened by genomic PCR for the deletion of the CSPG4 gene using the primers 5’-GGGCCCTTTAAGAAGGTTGA-3’ and 5’-GTTTTGACAGCCCAAACCAG-3’. Cell lines were further screened by immunoblotting and flow cytometry to verify the knockdown efficiency of the CSPG4 protein.

### Immunoblotting

Immunoblotting was performed using our standard protocol as described (30). Briefly, cell lysates were prepared and separated on 7.5–12% SDS-polyacrylamide gels and transferred to polyvinylidenedifluoride (PVDF) membranes. The membranes were blocked with 5% milk blocking solution in TBST and incubated overnight at 4°C with the respective primary antibodies. After several washes with TBST, the membranes were subsequently incubated with HRP-conjugated secondary antibody and the signals were detected by the ECL substrate (ThermoScientific, Waltham, MA, USA).

### Flow Cytometry

The cells were released in phosphate buffered saline (PBS)/ethylenediamine tetraacetic acid (EDTA) solution and washed 2 times with FACS buffer (RPMI medium supplemented with 1% goat serum and 5 mM HEPES). The cells were incubated with the indicated primary antibody for 45 min at 4°C, washed 3 times with FACS buffer, and then incubated with species-matched phycoerythrin-conjugated secondary antibody for 30 min at 4°C. The cells were analyzed on a BD Biosciences Accuri C6 Flow Cytometry System.

### Colony Formation Assay

For 3D colony formation assay, a layer of 1% agarose in regular growth medium was pipetted into six well plates and allowed to solidify. The cells were resuspended in 6.75 ml regular growth medium at 5,000 cells/ml and incubated for 15 min at 37°C. 750 μl of 2% agarose was then added to the tubes, mixed thoroughly by pipetting, and 2 ml of cell suspension was pipetted into triplicate wells. The plates were placed at 4°C for 15 min to facilitate rapid polymerization of the agarose, and the wells overlaid with 2 ml growth medium and incubated at 37°C/5% CO_2_ for 12 days. The medium was replaced every three days. The colonies were counted and the data were expressed as the average number (± s.e.m) of colonies from five fields/well from triplicate wells.

### Cell Invasion Assay

The cells (2.5 × 10^4^) in normal growth medium were added to the top chamber of triplicate wells of matrigel invasion chambers (8 μm, Corning, NY, USA), the bottom chambers filled with complete growth medium and cultured for 24 h at 37°C/5% CO_2_. The remaining cells in the upper chamber were removed with a cotton swab and the invaded cells fixed and stained using Differential Quick Staining Kit (Electron Microscopy Sciences, Hatfield, PA, USA). The invaded cells were enumerated under a microscope at ×100 magnification from five random fields/well. The data shown are the average number (± s.e.m) of invaded cells from five fields/well from 3 combined experiments. Statistical significance was determined using Students t-test.

### Co-Immunoprecipitation Assay

For co-immunoprecipitation experiments, the cells were lysed on ice with IP buffer (20 mM Tris–HCl, pH 7.5, 150 mM NaCl, 1 mM Na_2_EDTA, 1 mM EGTA, 1% Nonidet P-40, 2.5 mM sodium pyrophosphate,1 mM glycerophosphate, 1 mM Na_3_O_4_, 1 μg/ml leupetin, 1 mM PMSF) and the insoluble materials were removed by centrifugation. The lysates were pre-cleared with protein A/G Sepharose beads (Amersham Pharmacia, Piscataway, NY) for 30 min at 4°C. The lysates were incubated with each antibody overnight at 4°C, and the immunocomplexes collected by incubation with protein A/G-Sepharose beads for 1 h at 4°C. The immunocomplexes were washed three times with lysis buffer at 4°C and the bead-associated proteins resolved by SDS-PAGE.

### Statistical Analysis

To analyze the demographic and clinicopathological parameters, categorical and continuous variables were expressed as counts (percentages) and mean ± s.d., respectively. In order to compare the differences of symmetrical distribution between continuous variables, t-test was used. Kaplan–Meier (KM) analysis and bilateral Logrank test were used to evaluate the effect of CSPG4 expression on OS. Patients without OS information were censored at the last follow-up date. Cox proportional hazards model was used to evaluate the risk factors for the treatment outcomes, which was expressed as hazard ratio (HR) and 95% confidence interval (CI). All statistical analyses were performed using SAS 9.4 software (SAS Institute Inc., NC, USA) and R 3.6.2 (https://www.r-project.org). All hypothesis tests were two-sided. The significance level was 0.05, and the marginal significance level was 0.15.

## Results

### *TP53* Aberrations Increase Tumor Mutation Burden in TNBCs

**Figure 1:**
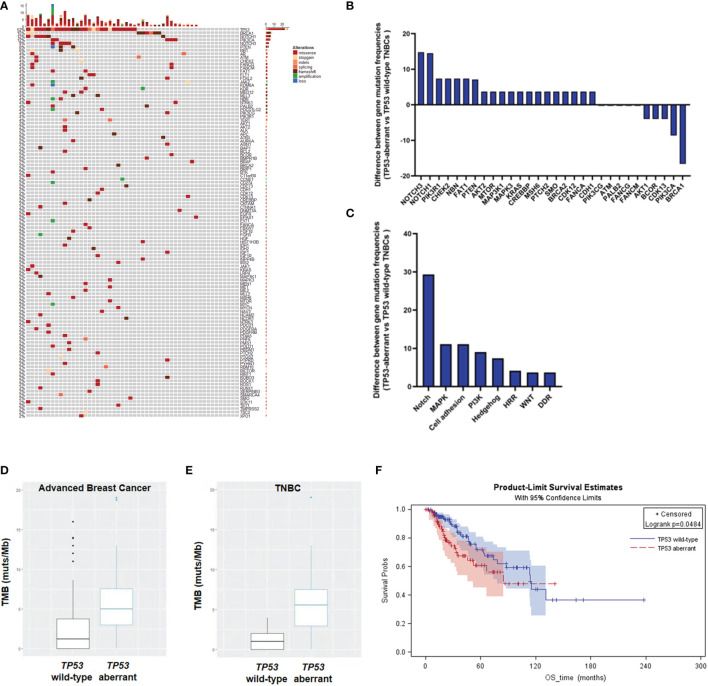
Heatmap and KM curves of *TP53* alterations and tumor mutation burden (TMB) in TNBCs. **(A)** Heatmap of *TP53* alterations in mTNBCs. **(B)** Difference of gene mutation frequencies between *TP53*-aberrant and *TP53* wild-type mTNBCs. **(C)** Difference of signaling pathways between *TP53*-aberrant and *TP53* wild-type mTNBCs. **(D, E)** Difference of TMB between *TP53*-aberrant and *TP53* wild-type metastatic breast cancer **(D)** and TNBC patients **(E)**. **(F)** KM curves for overall survival (OS) between *TP53*-aberrant and *TP53* wild-type breast cancer patients.

We have previously demonstrated that more than 30% of metastatic breast cancers had *TP53* aberrations (9). In this study, NGS analysis on ctDNA revealed that about 52% (27/52) of mTNBCs had *TP53* aberrations (**Figure 1A**). The gene aberrations and pathway enrichment in *TP53*-aberrant and *TP53* wild-type mTNBCs showed distinct genetic landscapes. mTNBCs with gene aberrations in Notch, MAPK, cell adhesion, PI3K, and Hedgehog pathways were strongly associated with *TP53* aberrations (**Figures 1B, C** **)**.

It has been demonstrated that high tumor mutation burden (TMB) is an important predictor for the treatment outcomes of PDL1 inhibition in lung cancer (31, 32) and colorectal carcinoma (33). Here, we found that *TP53*-aberrant metastatic breast cancers had significantly higher TMB than *TP53* wild-type metastatic breast cancers (median: 5.00 muts/Mb *vs* 1.44 muts/Mb; *P* = 0.004) (**Figure 1D**). In addition, *TP53*-aberrant mTNBCs had significantly higher TMB than *TP53* wild-type mTNBCs (median: 5.56 muts/Mb *vs* 1.00 muts/Mb; *P* = 0.0001) (**Figure 1E**). This trend was not significant in non-TNBCs (**Supplementary Figure 1**).

In addition, our previous study has suggested *TP53* aberrations to be a significant risk factor for PFS in metastatic breast cancer (9). In this study, KM curves derived from the TCGA-BRCA dataset showed that advanced breast cancer patients with *TP53* aberrations had poorer OS, compared with patients with wild-type *TP53* (*P* = 0.0484) (**Figure 1F**). These results suggest that *TP53* aberrations could lead to genomic instability and significantly increased tumor mutation loads in mTNBCs, which might be associated with the poor clinical outcomes in TNBC.

### CSPG4 and PDL1 Are Highly Expressed in *TP53*-Aberrant TNBCs

**Figure 2:**
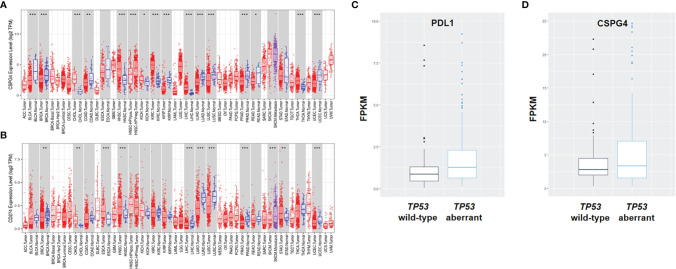
Expression of PDL1 and CSPG4 in different types of human cancers and subtypes of breast cancer. **(A, B)** The expression levels of human PDL1 **(A)** and CSPG4 **(B)** in different tumor types and subtypes of breast cancer analyzed using the TIMER2.0 database. **(C, D)** The expression levels of PDL1 **(C)** and CSPG4 **(D)** between *TP53*-aberrant and *TP53* wild-type TNBC patients. *P < 0.05, **P < 0.01, ***P < 0.001.

Since higher TMB is a predictor of the efficacy of PDL1-targeted immunotherapy in cancer, we speculated that the increased *TP53* aberrations would lead to increased expression of PDL1 in TNBC. To this end, we analyzed the mRNA expression levels of PDL1 in human cancers using the TIMER2.0 database. Indeed, the mRNA level of PDL1 was higher in basal-like breast cancer (BLBC)/TNBC than luminal-type breast cancer (**Figure 2A**). Because of the inefficiency of PDL1-targeted immunotherapy in most TNBC patients and the major role for CSPG4 has been shown to be overexpressed in TNBC, we though co-expression we investigated the status of CSPG4 in TNBC in this study. The high expression level of CSPG4 in TNBC was confirmed through analyzing the TCGA-BRCA dataset, showing that CSPG4 expression was higher in BLBC/TNBC than other subtypes of breast cancer (**Figure 2B**).

To further assess the role of *TP53* aberrations in the expression of CSPG4 and PDL1, we analyzed the transcriptome profile of breast cancers in the TCGA database. As shown in **Figure 2C**, PDL1 was significantly higher in *TP53*-aberrant TNBCs compared with *TP53* wild-type TNBCs (median: 1.29 *vs* 0.86, *P* = 0.01). As for CSPG4, there was an obvious trend of increased level in *TP53*-aberrant TNBCs compared with *TP53* wild-type TNBCs (median: 3.60 *vs* 3.29, *P* = 0.47), although the difference was not statistically significant (**Figure 2D**). These findings suggest that *TP53* aberrations might be associated with the increased TMB level and high expression of CSPG4 and PDL1 in mTNBCs and that CSPG4 might be used as an alternative or supplementary target for the therapeutic intervention in TNBC.

### CSPG4 and PDL1 Are Highly Correlated in Advanced TNBC Tissues and TNBC Cell Lines

**Figure 3:**
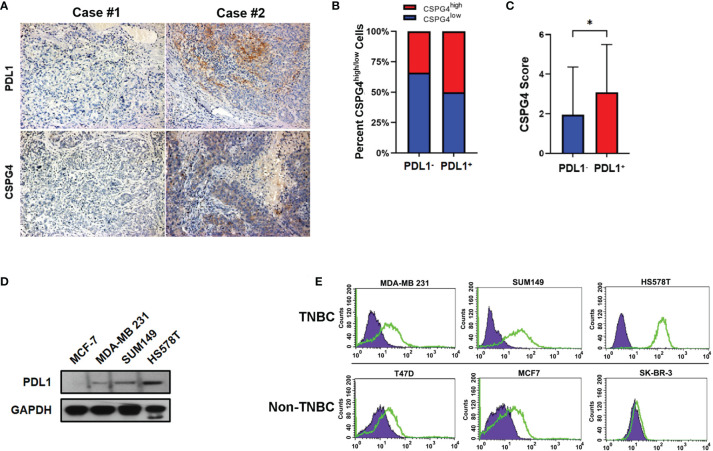
Expression of PDL1 and CSPG4 in advanced TNBC tissues and breast cancer cell lines. **(A)** Representative IHC images of PDL1 and CSPG4 protein expression in advanced TNBC tissues. The upper-left and upper-right panels show PDL1^−^ and PDL1^+^ tissue, respectively; and the lower-left and lower-right panels show CSPG4^−^ and CSPG4^+^ tissue, respectively. Original magnification: ×400. **(B)** Bar plot comparing the percentage of CSPG4^high^ tissues in PDL1^−^ and PDL1^+^ advanced TNBC samples. **(C)** Bar plot comparing the staining score of CSPG4 in PDL1^−^ and PDL1^+^ advanced TNBC samples. **(D)** PDL1 expression in breast cancer cell lines detected by immunoblotting assay. **(E)** CSPG4 expression in breast cancer cell lines detected by flow cytometry. (Purple area represents isotype-matched antibody staining; Green area represents CSPG4 staining). *P < 0.05.

Considering the important role of CSPG4 and PDL1 in malignant proliferation, metastasis and immunosuppression and their over-expression in *TP53*-aberrant TNBCs, we further examined the expression levels of CSPG4 and PDL1 in tissue samples from advanced TNBCs using IHC staining. IHC assays demonstrated that the positive rate of CSPG4 and PDL1 in advanced TNBC samples were about 60 and 35%, respectively. We further asked whether CSPG4 was associated with PDL1 expression in TNBC. Interestingly, we found a majority of TNBC patients with high CSPG4 expression also had high PDL1 expression. **Figure 3A** shows representative TNBC cases who had low levels of both PDL1 and CSPG4 (left) and high levels of both PDL1 and CSPG4 (right). Quantification of the IHC results revealed that the percentage of CSPG4^high^ TNBC patients (CSPG4 expression score ≥4) was higher in PDL1-positive patients than PDL1-negative patients (50% *vs* 34%, *P* = 0.144) (**Figure 3B**). Compared with PDL1-negative TNBCs, PDL1-positive TNBC samples had significantly higher CSPG4 expression level (scores) (3.09 *vs* 1.96, *P =* 0.0368) (**Figure 3C**). These findings suggest that CSPG4 and PDL1 were highly co-expressed in TNBC tissues.

To further investigate the expression of PDL1 and CSPG4 in TNBC cells, we analyzed their protein levels in several breast cancer cell lines by immunoblotting and flow cytometry. **Figure 3D** shows that, compared with ER-positive MCF-7 cells, TNBC cell lines SUM149, MDA-MB 231 and HS578T had significantly higher PDL1 levels. As shown in **Figure 3E**, compared with ER-positive (T47D and MCF-7) and HER2-positive (SK-BR-3) cell lines, the expression of CSPG4 was significantly higher in TNBC cell lines SUM149, MDA-MB 231, and HS578T. All these TNBC cells examined so far had *TP53* mutations. For example, MDA-MB-231 cells had *TP53* p.P72R mutation (34); SUM149 cells had *TP53* p.M237I mutation (35); HS578T cells had *TP53* V157F mutation (36). Furthermore, the TNBC cell line HS578T that had the highest level of PDL1 (**Figure 3D**) also had the highest level of CSPG4 (**Figure 3E**). These findings suggest that both CSPG4 and PDL1 were highly expressed and the expression of these two proteins was positively correlated in TNBC cells.

### Co-Expression of CSPG4 and PDL1 Has Important Prognostic Value in TNBCs

**Figure 4:**
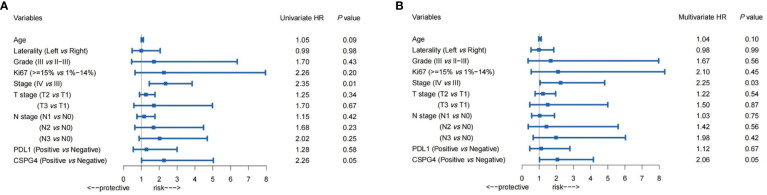
Prognostic value of PDL1, CSPG4, and clinicopathological variables in advanced TNBCs. Univariate **(A)** and Multivariate **(B)** Cox regression analysis showed the hazard ratios (HRs) (with 95% CI) of PDL1, CSPG4, and clinicopathological variables on progression-free survival (PFS) in advanced TNBCs.

In TNBC samples, both univariate and multivariate Cox regression analyses were performed to evaluate the risk factors for progression-free survival (PFS) in advanced TNBCs. As shown in **Figure 4**, in univariate and multivariate Cox regression analyses, the hazard of progression for PDL1-positive patients was 1.28 times and 1.12 times, respectively, higher than PDL1-negative patients (95% CI: 0.54–3.00, univariate; 0.45–2.79, multivariate), but not statistically significant. However, the progression of CSPG4-positive patients was significantly higher than that of CSPG4-negative patients, with an HR of 2.26 (95% CI: 1.01–5.03, *P* = 0.05) and 2.06 (95% CI: 1.02–4.15, *P* = 0.05), respectively, in univariate and multivariate Cox regression analyses. Therefore, both univariate and multivariate Cox regression analyses indicate that CSPG4 positivity is an important adverse prognostic factor for advanced TNBC.

**Figure 5:**
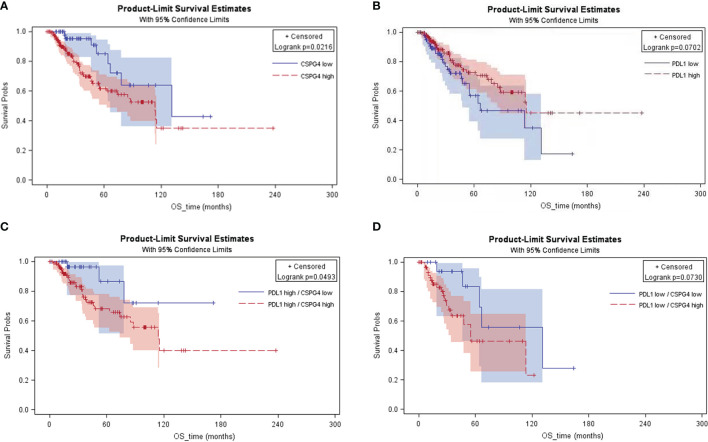
KM curves of the advanced breast cancer patients with different expression levels of CSPG4 and PDL1 on overall survival. **(A)** Survival rate between CSPG4^high^ and CSPG4^low^ advanced breast cancer patients. **(B)** Survival rate between PDL1^high^ and PDL1^low^ advanced breast cancer patients. **(C)** Survival rate between CSPG4^high^ and CSPG4^low^ patients in PDL1^high^ advanced breast cancer patients. **(D)** Survival rate between CSPG4^high^ and CSPG4^low^ patients in PDL1^low^ advanced breast cancer patients.

The effect of CSPG4 in conjugation with PDL1 overexpression on patient survival was further analyzed in advanced breast cancers by using the TCGA-BRCA database. The higher expression of CSPG4 was correlated with poorer OS (*P* = 0.0216) (**Figure 5A**). However, PDL1 high level did not show significant risk to OS; instead, PDL1 high level was a marginally protective factor for OS (*P* = 0.0702) (**Figure 5B**). When combined with PDL1, CSPG4 high level was still a significant risk factor for OS. Among the PDL1^high^ advanced breast cancers, CSPG4 high level was a significant risk factor for poor OS (*P* = 0.0493) (**Figure 5C**). Among the PDL1^low^ advanced breast cancers, CSPG4 high level was also a marginally significant risk factor for poor OS (*P* = 0.0730) (**Figure 5D**). These results suggest that co-expression of CSPG4 and PDL1 had important prognostic value in advanced breast cancers.

### EMT-Related Pathways Are Enriched in TNBCs With High Expression of CSGP4 and PDL1

**Figure 6:**
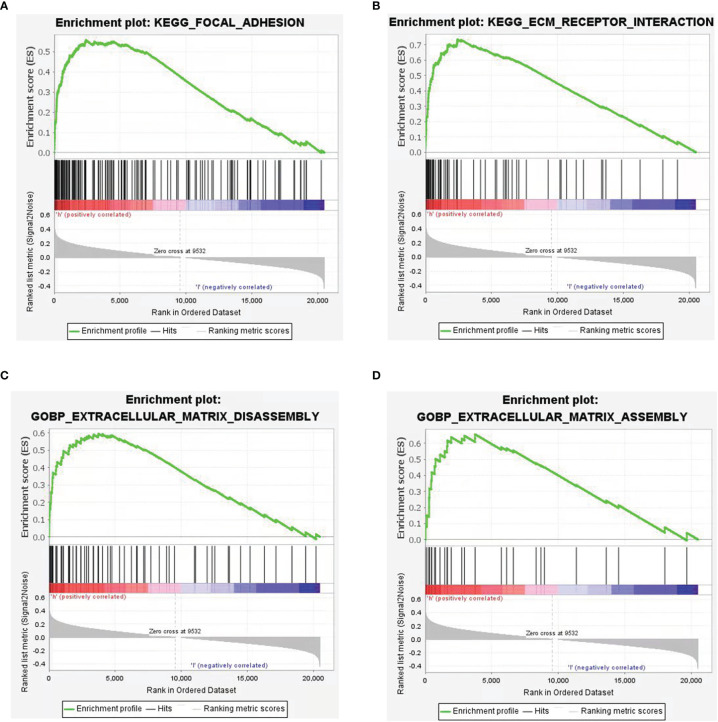
Gene Set Enrichment Analysis (GSEA) according to CSPG4 expression level in TNBCs. **(A–D)** Significant enrichment plots of EMT-related pathways in CSPG4^high^ TNBCs using GSEA, namely, focal adhesion **(A)**, extracellular matrix receptor interaction **(B)**, extracellular matrix disassembly **(C)**, extracellular matrix assembly **(D)**.

In order to screen the potential biological pathways that were related with the expression of CSPG4/PDL1 in TNBC, we performed GSEA comparing between the high and low CSPG4 and PDL1 expression groups. Gene sets with *P <*0.05 and FDR <0.25 were considered as significantly enriched. As shown in **Figure 6**, EMT-related pathways, namely, focal adhesion, extracellular matrix receptor interaction, extracellular matrix disassembly, extracellular matrix assembly, regulation of actin cytoskeleton were all significantly enriched in TNBC with CSPG4^high^ expression. In TNBC with PDL1^high^ expression, cell adhesion molecules were also significantly enriched (**Supplementary Figure 2**). To further investigate the relationship between CSPG4 or PDL1 and EMT, we constructed a PPI network between these two proteins and their correlated proteins by using the STRING v.11.0. As shown in **Supplementary Figure 3**, we found close correlations between CSPG4 and the EMT-related proteins, namely, SDC1, HSPG2, ITGB1, etc. PDL1 was also correlated with EMT-related proteins, namely, PTPN11, PXN, VAV1, etc. (**Supplementary Figure 4**). These data provide a functional link between CSPG4 expression and the EMT-related pathways.

**Figure 7:**
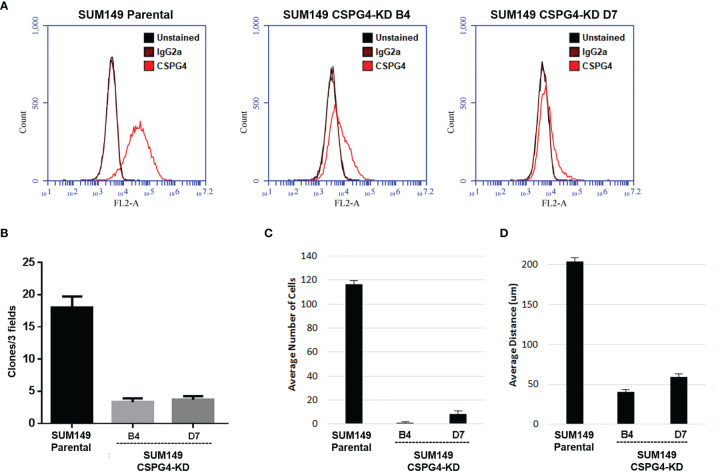
CSPG4 knockdown changes several EMT markers, implicating to reverse the mesenchymal phenotype. **(A)** Establishment of CSPG4-knockdown breast cancer cell lines B4 and D7 using CRISPR/CAS9 technology. **(B–D)** Colony formation numbers **(B)**, invasive cell numbers **(C)**, and the invasive distance **(D)** in established CSPG4-knockdown cell lines.

In order to further investigate the effect of CSPG4 on the EMT phenotype in TNBC cells, we knocked down CSPG4 expression using the CRISPR/CAS9 technology. As shown in **Figure 7A**, by using CRISPR/CAS9-mediated gene silencing, we successfully suppressed the CSPG4 level in TNBC SUM149 cells, and constructed SUM149-CSPG4-CRISPR-B4 and SUM149-CSPG4-CRISPR-D7 cell lines. In these SUM149-CSPG4-knockdown cells, the colony formation capability was significantly inhibited (**Figure 7B**). Both SUM149-CSPG4-CRISPR cell lines had significantly lower number of invasive cells and lower invasive distance (**Figures 7C, D**).

**Figure 8:**
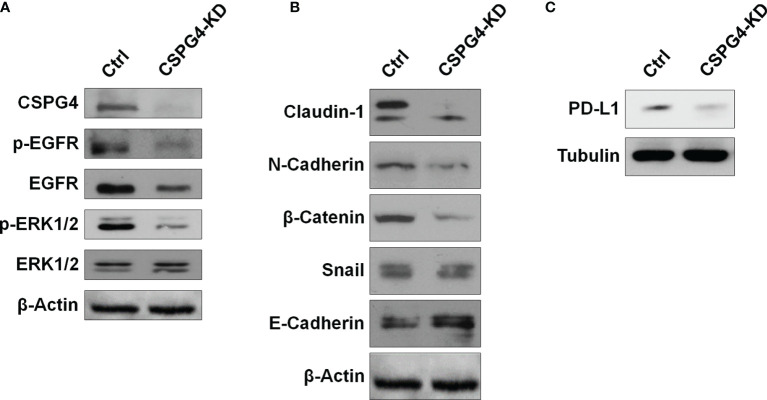
CSPG4 knockdown affects EGFR and ERK1/2 activation and PDL1 expression. The protein levels of phosphorylated and unphosphorylated EGFR and ERK1/2 **(A)**, EMT markers **(B)**, and PDL1 **(C)** in CSPG4-knockdown SUM149 cells.

Because EGFR/ERK1/2 signaling is important for the regulation of EMT-related markers, we examined the status of the EGFR/ERK1/2 signaling pathway in CSPG4-silenced TNBC cells. We found that both phosphorylated EGFR and phosphorylated ERK1/2 were inhibited in SUM149-CSPG4-knockdown cells (**Figure 8A**). The EMT markers Claudin-1, N-Cadherin, and β-Catenin were significantly inhibited (**Figure 8B**). EMT is reported to drive immune-suppression *via* the Zeb1 transcription factor, which induces the expression of PDL1 on these invading cells (37). We thus also checked the level of PDL1 in CSPG4-knockdown cells. **Figure 8C** showed that the expression of PDL1 was significantly inhibited in SUM149-CSPG4-knockdown cells. These findings suggested that CSPG4 might mediate PDL1 through EGFR/ERK1/2/EMT markers pathway in TNBC cells.

## Discussion

Since high TMB associated with the prevalent *TP53*-aberrations is an important predictor for the treatment outcomes of PDL1 blockade in cancer, immunotherapy becomes a potential option in TNBCs. However, despite the clinical benefits of PDL1/PD1 blockade in some TNBC patients, therapy resistance remains a significant challenge for further clinical application of PDL1/PD1-targeted immunotherapy. Therefore, other therapeutic targets are worth exploring, at least, in the context of enhancing the therapeutic efficacy of immune checkpoint inhibition. In this study, we found that CSPG4 was upregulated and co-expressed with PDL1 in TNBCs and that the high expression of CSPG4 was a significant prognostic factor for poor PFS and OS in advanced TNBCs. CSPG4 thus might provide a new target that can be coupled with and enhance the efficacy of PDL1/PD1-directed immunotherapy for TNBC.

As a scaffold protein, CSPG4 may not only bind to a variety of kinases and extracellular factors to mediate the activation of multiple signaling pathways (22), but also interacts with PDL1 on the surface of TNBC cells. In addition, CSPG4 might be able to induce PDL1 expression through the SNAI1/SIRT3 pathway (38). SNAI1 and ZEB1 upregulated PDL1 by binding directly to E-boxes in PDL1 promoter region (39). In addition, by stabilizing SNAIL and inhibiting AXIN2, SIRT1 upregulates PDL1 by enhancing the binding of beta-catenin/TCF to PDL1 promoter region (40). Consistent with above research findings, we found that the knockdown of CSPG4 could significantly inhibit the phosphorylated-EGFR and beta-catenin, and thus suppressed PDL1 level in TNBC cells. Based on these findings, we supposed that CSPG4 overexpression facilitated PDL1 expression through EMT-related pathways.

In survival analysis, we found CSPG4 to be a significant risk factor for poor response to 1st-line chemotherapy in advanced TNBCs. High expression of CSPG4 promotes tumor cell proliferation, angiogenesis, immune escape, and therapy resistance (21). We found that high level of CSPG4 was also a significant risk factor for OS in advanced breast cancers, suggesting a critical role for CSPG4 in determining the outcomes of advanced breast cancers. However, how CSPG4 leads to adverse clinical outcomes of advanced breast cancers is still not known and will be an interesting topic to investigate. Interestingly, we found that knocking down of CSPG4 by CRIPR/CAS9-mediated gene silencing led to downregulation of PDL1 (data not shown), suggesting a mechanistic link between these two cell surface molecules. Further studies will be needed to elucidate through which intracellular signaling pathway(s) CSPG4 is linked to PDL1, thus impacting the clinical outcomes of advanced TNBCs.

As TNBC-specific cell surface antigens, CSPG4 and PDL1 have a potential targeted therapeutic value. Because of their potential molecular mechanism of interaction, targeting either molecule may not achieve complete tumor regression, highlighting the necessity of co-targeting both molecules. Our results justify CSPG4 as a valid therapeutic target that might be used in conjugation with PDL1-targeted strategy in TNBC. This study will provide clues and call for further exploring the therapeutic value of CSPG4 and PDL1 in TNBC.

## Data Availability Statement

The original contributions presented in the study are included in the article/**Supplementary Material**. Further inquiries can be directed to the corresponding authors.

## Ethics Statement

This study was approved by the ethics committee of Hunan Cancer Hospital with the approval number of kyjj-2019-014. The author declares that he has received ethical recognition. The authors declare that they have obtained ethical approval and patients consent to participate. The patients/participants provided their written informed consent to participate in this study.

## Author Contributions

ZH, CZ, JY, SD and CT performed the experiments. NX, LX, MW and SF collected and analyzed the data. ZH, CZ and JY drafted the manuscript. ZR, MP and JM reviewed the manuscript. QO, JL and XD conceived and designed the research. All authors listed have made a substantial, direct, and intellectual contribution to the work and approved it for publication.

## Funding

This work was supported by the National Natural Science Foundation of China (81872167, 82173374, 82103342), the Key Grant of Research and Development in Hunan Province (2018SK2124, 2020DK2002), the Natural Science Foundation of Hunan (2020JJ8064, 2019JJ50360, 2019JJ40193, 2020JJ5386), the Hunan Provincial Health Commission Project (B2019089, C2019070), and the Changsha Science and Technology Project (kq2004125).

## Conflict of Interest

The authors declare that the research was conducted in the absence of any commercial or financial relationships that could be construed as a potential conflict of interest.

## Publisher’s Note

All claims expressed in this article are solely those of the authors and do not necessarily represent those of their affiliated organizations, or those of the publisher, the editors and the reviewers. Any product that may be evaluated in this article, or claim that may be made by its manufacturer, is not guaranteed or endorsed by the publisher.
