# Supplementary material for: Co-Expression and Combined Prognostic Value of CSPG4 and PDL1 in TP53-Aberrant Triple-Negative Breast Cancer
Source: Front Oncol. 2022 Feb 24;12:804466. doi: 10.3389/fonc.2022.804466 (PMC8907582; doi:10.3389/fonc.2022.804466)
Supplement: Supplementary file 1 [file DataSheet_1.docx]

**Supplementary Tables and Figures**

**Supplementary Table 1**. **The clinicopathological characteristics of advanced TNBC patients**

| **Covariates** | **Level** | **All patients (n=85)** |
| --- | --- | --- |
| Age at diagnosis |  | 47.27 ± 7.98 |
| Laterality | Right | 46 (54.12%) |
|  | Left | 39 (45.88%) |
| Histopathological grade | 2–3 | 22 (25.88%) |
|  | 3 | 63 (74.12%) |
| Histopathological type | Invasive ductal carcinoma | 69 (80.23%) |
|  | Invasive lobular carcinoma | 7 (8.14%) |
|  | Invasive carcinoma, NOS | 8 (9.30%) |
|  | Carcino-sarcoma | 1 (1.16%) |
|  | Paget’s disease | 1 (1.16%) |
| Stage | III | 37 (43.53%) |
|  | IV | 48 (56.47%) |
| T stage | T1 | 21 (24.71%) |
|  | T2 | 59 (69.41%) |
|  | T3 | 5 (5.88%) |
| N stage | N0 | 45 (57.65%) |
|  | N1 | 19 (22.35%) |
|  | N2 | 14 (16.47%) |
|  | N3 | 7 (8.23%) |
| Ki67 | 10–14% | 7 (8.24%) |
|  | 15–69% | 49 (57.65%) |
|  | ≥70% | 29 (34.12%) |

**
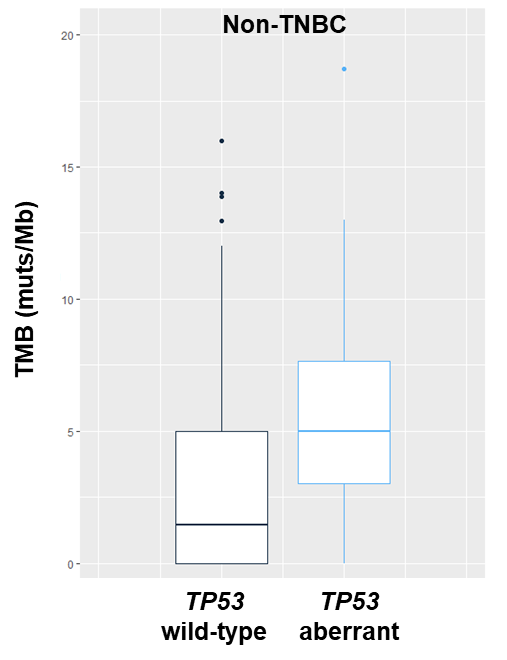
**

**Supplementary Figure 1. Tumor mutation burden (TMB) in non-TNBCs.** Difference of TMB between *TP53*-aberrant and *TP53* wild-type non-TNBCs.

**
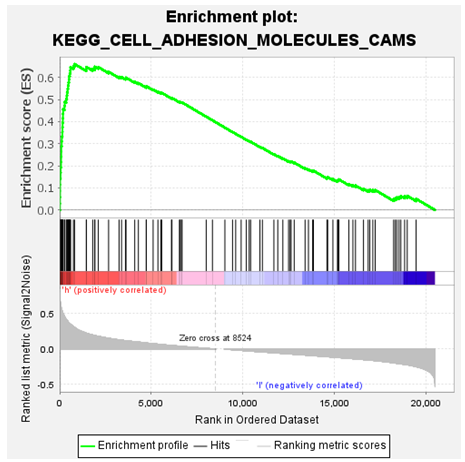
**

**Supplementary Figure 2. Gene Set Enrichment Analysis (GSEA) according to the PDL1 expression level in TNBCs**. Significant enrichment plots of the pathway related to cell adhesion molecules in PDL1^high^ TNBCs using GSEA.

**
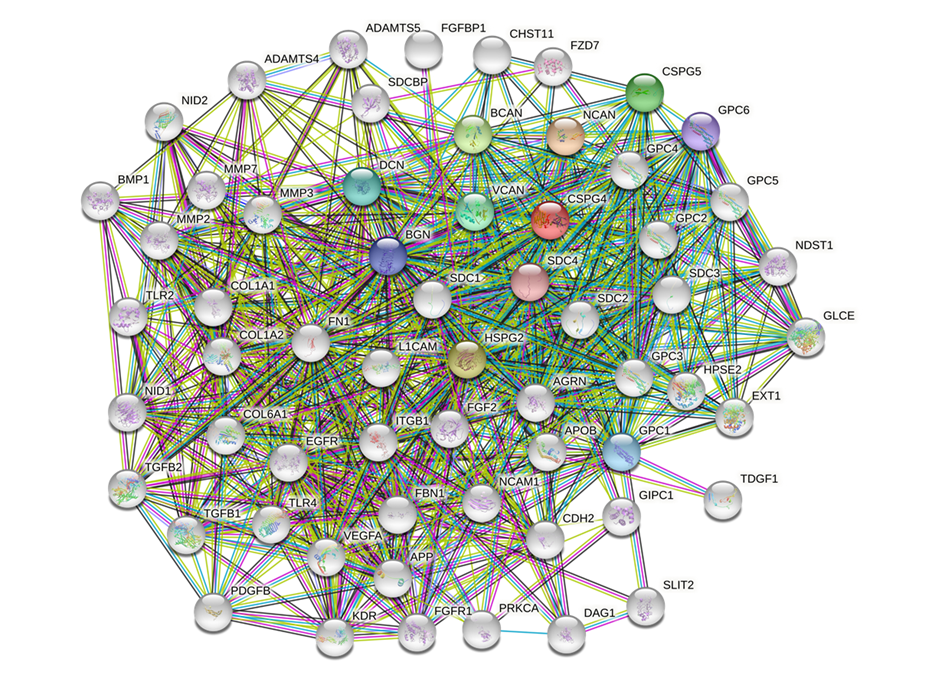
**

**Supplementary Figure 3**. **Protein-protein interaction (PPI) network of CSPG4 and their correlated proteins.** PPI network of CSPG4 and their correlated proteins by using the STRING v.11.0.

**
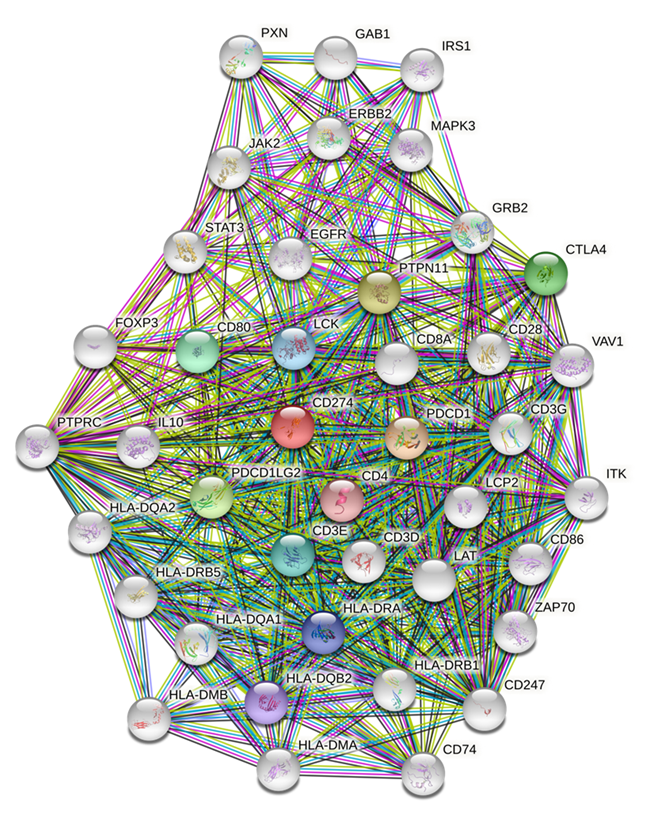
Supplementary Figure 4. Protein-protein interaction (PPI) network of PDL1 and their correlated proteins.** PPI network of PDL1 (CD274) and their correlated proteins by using the STRING v.11.0.
